# Prediction of post-treatment retinal sensitivity by baseline retinal perfusion density measurements in eyes with branch retinal vein occlusion

**DOI:** 10.1038/s41598-020-66708-0

**Published:** 2020-06-15

**Authors:** Soraya Rachima, Kazutaka Hirabayashi, Akira Imai, Yasuhiro Iesato, Toshinori Murata

**Affiliations:** 1 Department of Ophthalmology, Diponegoro University, Semarang-Central Java, Indonesia; 2 Department of Ophthalmology, Shinshu University School of Medicine, Nagano, Japan

**Keywords:** Eye diseases, Retinal diseases

## Abstract

In this study, we investigated the longitudinal correlation between macular sensitivity and perfusion density (PD) in retinas affected by branch retinal vein occlusion. Retinal sensitivity was measured using microperimetry and PD was measured by optical coherence tomography angiography. We also investigated the possibility that the PD, 1 month after anti-vascular endothelial growth factor (VEGF) treatment, is a predictor of retinal sensitivity after 1 year of successful macular oedema management with anti-VEGF. The correlation between measurements of retinal sensitivity and PD at baseline (1 M) and at 6 and 12 months were investigated. There was a significant positive correlation between retinal sensitivity and PD at all time points (baseline (1 M), r = 0.67, *P* < 0.0001; 6 months, r = 0.59, *P* < 0.0001; 12 months, r = 0.62, *P* < 0.0001) and between the PD at 1 month and retinal sensitivity at 12 months (r = 0.63, *P* < 0.0001). Unlike in areas that showed a mild to moderate decline in PD, retinal sensitivity in areas where the decrease in PD was severe at baseline did not show significant improvement with treatment over time. These findings suggest that the PD value measured using optical coherence tomography angiography at or soon after the baseline can predict retinal sensitivity after 1 year of anti-VEGF treatment.

## Introduction

Retinal vein occlusion (RVO) is a common cause of retinal vascular disease. It can be classified according to the anatomical location and retinal ischaemia and has multiple predisposing and precipitating factors^1–3^. One subtype is the branch retinal vein occlusion (BRVO), which is thought to be caused by thrombosis at the level of the branch retinal vein where it is crossed by a sclerotic artery^4^. Occlusion of this vein leads to disturbance of haemodynamics and vascular autoregulation, resulting in retinal haemorrhage, venous dilatation, macular oedema, serous retinal detachment, and intraocular neovascularisation^5,6^.

In approximately 80% of cases, BRVO is accompanied by macular oedema. One-third of patients show spontaneous resolution of macular oedema; however, if left untreated, BRVO can cause permanent impairment of vision in the residual patients^7–9^.

Although the underlying cause of macular oedema has not been clearly established, some hypotheses have been advocated. Accumulation of fluid in macular oedema seems to be associated with a response to vascular endothelial growth factor (VEGF) derived from the hypoxic retinal tissue^10,11^. Several anti-VEGF agents are available to attenuate this pathology and can significantly reduce macular oedema and improve vision^12–14^. The effectiveness of ranibizumab has been confirmed in several studies, including BRAVO study^15^.

Non-invasive diagnostic tools, including the optical coherence tomography angiography (OCTA), have been developed to allow us to perform an instant three-dimensional vascular mapping of the retina and choroidal vascularisation without the need for dye injection^16,17^. OCTA is also more useful than the fluorescein angiography which has a limited ability to capture the topography and condition of the vessels accurately, not only because of the intraretinal haemorrhages during the acute stage of the disease but also due to the fluorescein dye leakage^18^. OCTA has enabled clinicians to determine macular vessel density in the superficial and deep retinal layers, and to quantitatively evaluate the areas with microvascular dropout at any stage of the disease^19,20^.

It has been reported that ranibizumab improves overall retinal sensitivity in the affected retina as well as visual acuity in patients with BRVO^21^. In a study using fluorescein angiography, retinal sensitivity was lower in the retinal non-perfusion areas^22^. If the macular vascular density measured by OCTA at an early stage of treatment can predict retinal sensitivity after treatment, it would be useful for explaining the prognosis to patients when starting the treatment. In this study, the retinal sensitivity and vessel density were compared in a point by point manner, but not as an overall average of the affected retina by BRVO. Their longitudinal correlation was investigated in the affected retina where macular oedema was well treated by anti-VEGF injections. We also investigated whether vessel density measured by OCTA at the beginning of treatment showed a correlation with macular sensitivity after one year of treatment.

## Results

The final study group comprised of 4 men and 4 women aged 61–80 years. The 12 months mean number of ranibizumab injection was 4.4. Mean baseline retinal sensitivity was 9.6 dB and the perfusion density (PD) at 1 month after treatment was 28% (Table 1). At each time point, the total number of areas both the retinal sensitivity measurement points and OCTA measurement sites ranged from 119–136. For both retinal sensitivity and OCTA, 17 measurement points were used for each patient. The measurement range was 119–136; one person could not be measured for baseline retinal sensitivity.

**Table 1:** Baseline demographic and clinical characteristics of the patients.

Eyes/patients, n/n	8/8
Age, years	70 ± 2.0
Sex	
Male	4
Female	4
12 months number of intravitreal ranibizumab injections	4.4 ± 2.4
Perfusion density (1 M), %	28 ± 12
Retinal sensitivity, dB	9.6 ± 7.6

The data are presented as the mean ± standard deviation, or number, as appropriate.

There was a significant positive correlation between the retinal sensitivity and PD at all assessment points (retinal sensitivity at baseline and PD at 1 month, r = 0.67, *P* < 0.0001; 6 months, r = 0.59, *P* < 0.0001; 12 months, r = 0.62, *P* < 0.0001; Fig. 2A–C). There was also a significant positive correlation between the PD at 1 month and retinal sensitivity at 12 months (r = 0.63, *P* < 0.0001; Fig. 2D). The perfusion density was 28 ± 12 1.2 to 51, 27 ± 12 0.8 to 52, and 22 ± 14 0.4 to 51 at 1, 6, and 12 months, respectively. Retinal sensitivity was 9.6 ± 7.6, 0 to 23, 16 ± 9.3, 0 to 29, 17 ± 9.7, and 0 to 30 at baseline 6, and 12 months, respectively (mean ± standard deviation, and range).

**Figure 1:**
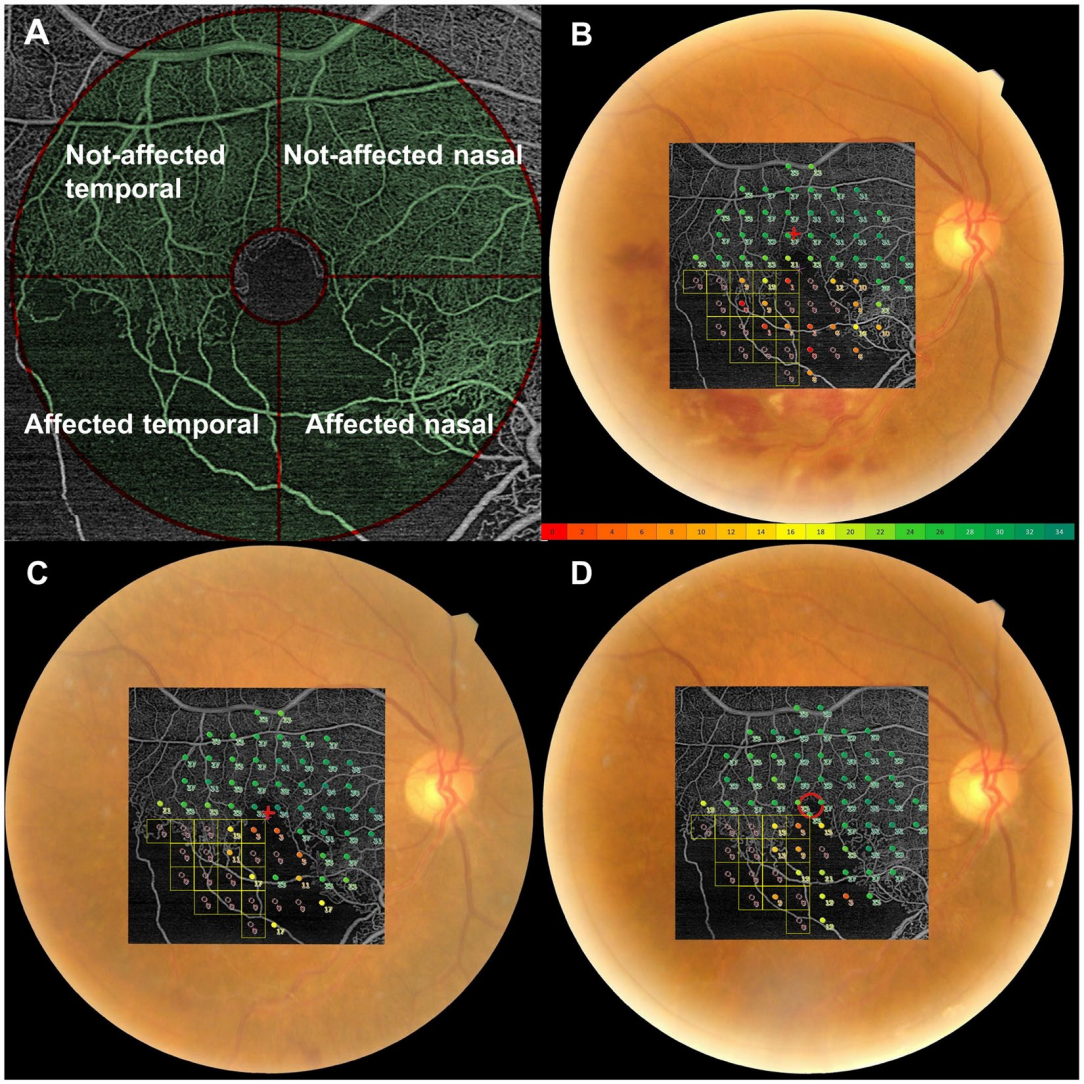
A representative case of a right lower branch retinal vein occlusion showing the optical coherence tomography angiography, fundus photograph images, and the change in retinal sensitivity. (**A**) Classification of the four quadrants. In this study, the affected temporal area was used for analysis. (**B**) Overlay of baseline optical coherence tomography angiography, fundus photograph, and retinal sensitivities. The yellow frames are the range where the perfusion density was measured, corresponding to each MP3 measurement point. (**C**) 6 months, (**D**) 12 months.

**Figure 2:**
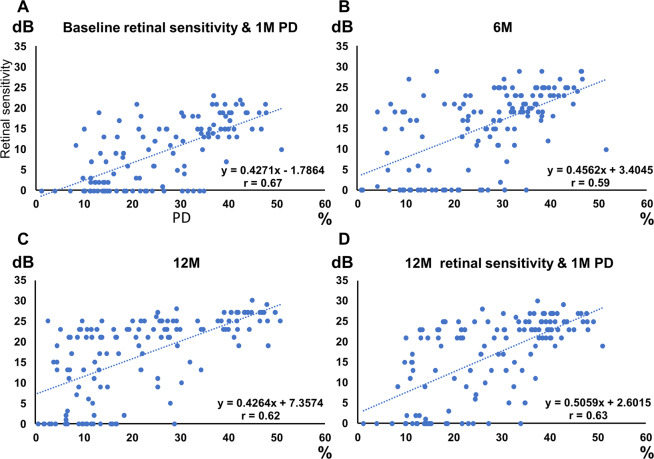
Correlation scatter plot of the retinal sensitivities and perfusion density at each time point. (**A**) Retinal sensitivity at baseline and PD at 1 month (r = 0.67, *P* < 0.0001), (**B**) 6 months (r = 0.59, *P* < 0.0001), (**C**) 12 months (r = 0.62, *P* < 0.0001), (**D**) correlation between retinal sensitivity at 12 months and the perfusion density at 1 month (r = 0.63, *P* < 0.0001). Significant positive correlations were found for (A–D). n = 119 at baseline, n = 136 at 6 and 12 months (D) using Pearson’s correlation coefficient.

**Figure 3:**
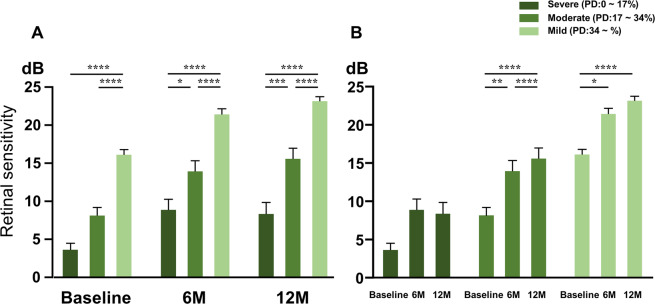
Comparison of mean retinal sensitivities at each time point in the three severity groups divided by perfusion density at 1 month: severe (0–17%), moderate (17–34%), or mild (34% or more). (**A**) Divided by time point. At all times, greater perfusion density was accompanied by higher retinal sensitivity. (**B**) No significant improvement was observed over time in the severe group divided by perfusion density but significant improvements were observed in the moderate and mild groups. (**A,B**) are rearrangements of the same data. The bars show the mean ± standard error of the mean (severe group: n = 35 at baseline, n = 36 at 6 and 12 months; moderate group: n = 42 at baseline, n = 45 at 6 and 12 months; mild group: n = 42 at baseline, n = 55 at 6 and 12 months). *P < 0.05, **P < 0.01, ***P < 0.001, ****P < 0.0001, from two-way analysis of variance with Tukey’s multiple comparisons test.

When each measurement area was categorised as severe, moderate, or mild depending on the PD status compared to the 1 month, the retinal sensitivity results were in the order of severe <moderate <mild at all evaluation period, i.e., baseline, 6 and 12 months (Fig. 3A). In the severe PD loss group, there was no significant difference in the retinal sensitivity between the baseline and 6 months, or baseline and 12 months (6 months, *P* = 0.0570; 12 months, *P* = 0.1297). In contrast, when compared to the baseline, the moderate and mild groups showed a significant improvement in the retinal sensitivity after 6 months (moderate group, *P* = 0.0054; mild group, *P* = 0.0101) and 12 months (moderate group, *P* < 0.0001; mild group, *P* < 0.0001; Fig. 3B). In the severe PD loss group retinal sensitivity was 3.6 ± 5.2, 8.9 ± 8.7, and 8.3 ± 9.2 at baseline, 6, and 12 months, respectively. In the moderate group the retinal sensitivity was 8.1 ± 7.0, 14 ± 9.3, and 16 ± 9.3 at baseline, 6, and 12 months, respectively. In the mild group the retinal sensitivity was 16 ± 4.4, 21 ± 5.4, and 23 ± 4.5 at baseline, 6, and 12 months, respectively (mean ± standard deviation; severe group: n = 35 at baseline, n = 36 at 6 and 12 months; moderate group: n = 42 at baseline, n = 45 at 6 and 12 months; mild group: n = 42 at baseline, n = 55 at 6 and 12 months).

**Figure 4:**
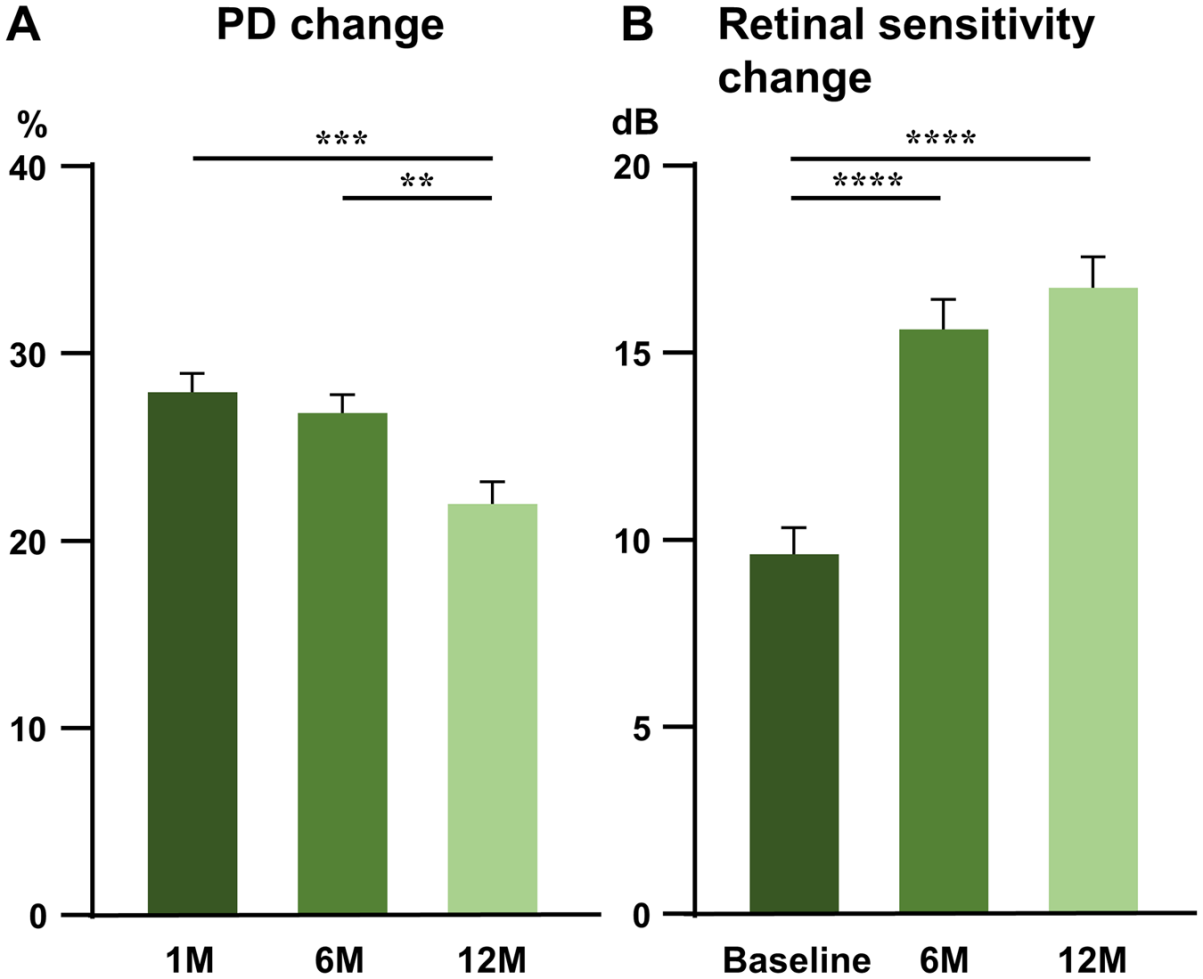
Change over time in all the average perfusion density and retinal sensitivities measured in the affected temporal area. (**A**) Change in perfusion density. Compared to 1 and 6 months, there was a significant decrease at 12 months (**B**) Change in retinal sensitivity. Compared to baseline, there was a significant improvement at 6 and 12 months. The bars show the mean ± standard error of the mean (n = 119–136 in each group). **P < 0.01, ***P < 0.001, ****P < 0.0001, one-way analysis of variance with Tukey’s post hoc test.

Average PD of all measurement points showed a significant decrease at 12 months compared to the 1 month (*P* = 0.0004; Fig. 4A). In contrast, mean retinal sensitivity showed a significant improvement at 6 and 12 months when compared to the baseline (both *P* < 0.0001; Fig. 4B). PD was 28 ± 12, 27 ± 12, and 22 ± 14 at 1, 6, and 12 months, respectively. Retinal sensitivity was 9.6 ± 7.6, 16 ± 9.3, and 17 ± 9.7 at baseline, 6, and 12 months, respectively (mean ± standard deviation). All these data followed a normal distribution.

## Discussion

In BRVO, the overall retinal sensitivity of the affected retina improved after successful treatment of macular oedema by anti-VEGF treatment^21^. In this study, we examined the point by point correlation between the retinal sensitivity and retinal circulation status evaluated by PD on OCTA. There was a positive correlation between the retinal circulation and retinal sensitivity in the corresponding areas. Since anti-VEGF therapy provides a much better vision than that of the natural innate healing process^15^, we hypothesized that anti-VEGF therapy may help with the survival of dying cells, which may lead to better retinal sensitivity and visual acuity. In addition, other factors such as the resolution of retinal oedema and haemorrhages after anti-VEGF therapy, which help vision recovery, may also be secondary to the retinal sensitivity gain in the area with low flow signals at the baseline. Consequently, we considered the possibility that the final retinal sensitivity may recover, even in the area of low vascular density at the baseline. However, we observed only little improvement in retinal sensitivity with time in the region where the PD loss was severe, while the sensitivity improved in the region where PD was moderate or mild. Therefore, we considered this was because severe PD loss resulted in more retinal neuronal cells death than moderate or mild PD loss. Overall average retinal sensitivity recovered since the affected retina included more areas with mild to moderate PD loss than those with severer PD loss. In measurement points with mild to moderate PD loss, the retinal sensitivity improved as retinal haemorrhage and oedema resolved after successful anti-VEGF treatment. In such areas, the major cause of macular sensitivity loss at baseline was haemorrhages and oedema, but not the neuronal cell death. In a study using fluorescein angiography, the retinal sensitivity remained low in the macular nonperfusion areas^22^.

Interestingly, we found a correlation between the PD obtained one month after the initial ranibizumab injection and retinal sensitivity after one year of anti-VEGF treatment, indicating that early PD measurements by OCTA are useful for predicting the prognosis of the final retinal sensitivity after successful macular oedema management for one year.

Like previous reports^23^, we considered that retinal sensitivity was lower in the affected areas with haemorrhage and oedema than in the areas free from such changes. Overall, it appears that anti-VEGF treatment improves the retinal sensitivity in BRVO by resolving haemorrhage and oedema, as long as retinal circulation in the corresponding area is preserved.

On the other hand, overall PD in the affected retina did not improve or even decrease in one year suggesting that the local reperfusion of the occluded vessels did not occur even after successful macular oedema management using the anti-VEGF treatment. In areas with mild or moderate PD loss, the resolution of macular haemorrhage and oedema lead to retinal sensitivity recovery, while it did not in the areas where the PD is significantly lowered.

These data suggest that the local retinal sensitivity after successful macular oedema treatment had already been determined, at least in part, at the onset of BRVO depending on the status of macular perfusion since the local PD did not improve with time in this study.

Tsai *et al*. reported that vessel density and retinal sensitivity also showed a correlation in diabetic retinas^24^. Based on this report and the results of the present study, we considered that macular sensitivity depends on the perfusion status in the corresponding areas in retinal vascular disorders such as BRVO and diabetic retinopathy.

It has also been reported that VEGF knockout mice do not complete angiogenesis, resulting in embryonic lethality^25^. VEGF is essential for the survival of vascular endothelial cells. Suppressing vascular permeability by anti-VEGF treatment may improve retinal oedema^26^ but compromises the survival of capillary vascular endothelial cells and thus may reduce PD.

There may be several reasons for the variation in the improvement of retinal sensitivity after treatment according to the retinal circulation status evaluated PD. If PD is maintained, retinal sensitivity in that area will increase with the resolution of haemorrhages and oedema. Retinal neuronal cells survive in these areas because of the oxygen and nutrient supply from the maintained retinal vessels. On the other hand, in areas where retinal capillaries are occluded (PD is low), the survival of retinal neuronal cells depends on the oxygen and nutrients in the oedema fluid that consists of serum leaked from remaining capillaries. Anti-VEGF treatment reduces macular oedema and improves vision, but this may also cause more damage to the retinal neuronal cells in the area where PD is low. Thus, overall retinal sensitivity improved in the affected area, but the point by point evaluation shows that the retinal sensitivity remains low in the area with low PD. If the oxygen supply from the retinal circulation is impaired and neuronal cells are damaged, the retinal sensitivity will not improve even if oedema and haemorrhage are resolved.

Provided that anti-VEGF treatment is available for a patient with BRVO and that macular oedema can be successfully suppressed, it is likely that there would be a correlation between retinal sensitivity at 12 months and PD evaluated one month after the first anti-VEGF injection when investigated in a point-by-point manner.

The limitations of this study are that only the eyes with major BRVO were included and that the number of patients was small. Other subtypes, including macular BRVO and BRVO that occurs in the optic disc^27^, were not investigated. Hayreh *et al*. reported that the prognosis of visual acuity differs between macular BRVO and major BRVO so that they are different entities, therefore, in this study only patients with major BRVO were enrolled^28^. In addition, the average retinal thickness in the measurement points where PD and retinal sensitivity were evaluated could not be measured in this experiment due to the limitations of the function of the measuring device. Therefore, the relationship between the retinal thickness and the PD or retinal sensitivity was not evaluated in a point-by-point manner. The investigation of the correlation between the haemorrhage and retinal sensitivity or between oedema and sensitivity was also not feasible.

OCTA evaluation of the foveal circulation using PD would be useful when explaining the vision prognosis to the patients since foveal sensitivity directly correlates with the central vision. As this study lacked controls, we cannot exclude the possibility of the natural/spontaneous healing; however, we observed an overall retinal sensitivity improvement after anti-VEGF treatment independent of the retinal perfusion improvement. In the future, substances that have the potential to induce reperfusion or physiologic retinal neovascularization into the nonperfusion areas may become therapeutic targets^29^. In addition to the treatment of retinal oedema and haemorrhage, development of a treatment to improve macular perfusion itself is warranted in future research.

## Patients and Methods

Patients with major BRVO who were treated at Shinshu University Hospital in 2017–2018, had MP3 measurement points recorded in the affected temporal region within 10 degrees of the fovea, and visited each month for 1 + pro re nata (PRN) intravitreal injections of ranibizumab 0.5 mg/0.05 ml (Lucentis kit for intravitreal injection, 10 mg/ml; Novartis, Basel, Switzerland) were identified and enrolled in the study. Patients with macular BRVO, who refused the injection, who failed to fulfil the monthly visit requirement, who had any ocular or periocular inflammation, and with artefacts in OCTA images precluding clear visualisation of the vessel were excluded. The foveal thickness was measured using OCT (CIRRUS HD-OCT; Carl Zeiss Meditec, Inc., Jena, Germany) as the average thickness of the retina in the 1 mm circle centred on the foveolar. After the first injection, ranibizumab was administered when the foveal thickness was found to be ≥300 μm measured by OCT at the time of a visit and when it increased by 20% over the thinnest foveal thickness. Swept-source OCTA (PLEX Elite 9000; Carl Zeiss Meditec, Inc.) was performed, and the retinal sensitivity (Microperimeter MP-3; Nidek Co., Ltd., Gamagori, Japan) for retinal sensitivity was measured at the time of the first injection and 6 and 12 months later. As described in the previous report, the baseline used for OCTA was one month after injection^30^. At 6 and 12 months, macular oedema was absent in all patients. The study was approved by the Shinshu University Institutional Review Board for Clinical Research and carried out in accordance with the tenets of the Declaration of Helsinki. Informed consent was obtained from all study participants.

### Optical coherence tomography angiography

En face swept-source OCTA images were obtained for all subjects using the PLEX Elite 9000. The scanning protocol covered a 6 × 6-mm field of view. Images were obtained at every visit, with the focus on those obtained at baseline and at the 6- and 12-month visits. Given that OCTA segmentation errors may be observed in patients with high levels of macular oedema and haemorrhage, an OCTA image obtained at 1 month after treatment was selected as the baseline image and corresponded to baseline retinal sensitivity. After obtaining a ‘raw’ OCTA image, we divided the calculation area into 4 regions, i.e., affected nasal, affected temporal, not affected nasal, and not affected temporal area in the whole retinal layers (from the internal limiting membrane to above the retinal pigment epithelium) (Fig. 1A, Supplementary Fig. S1). After the obtained image was binarized by local Otsu method using Fiji software (ImageJ 2.0.0-rc-69/1.52p)^31,32^, the PD was calculated for an area of 600 μm × 600 μm around each of the 17 measurement points of MP3 in the affected temporal region (Fig. 1B–D). The PD was defined as the ratio of the total area covered with vessels over the total calculation area (%). The 1 month PD was classified according to the degree of ischaemia as severe (0–17%), moderate (17–34%), or mild (34% or more). En face images were excluded if they had any of the following: signal strength lower than 6, motion artefacts, and visualisation of vessel impossible because of any media opacity. An eye-tracking system was used while obtaining OCTA images to minimise artefact. With a small amount of haemorrhage, OCTA captures a nonperfusion area closer to the macula than FA^18^. In 8 cases, we obtained high-quality OCTA images with signal strengths of 7 or more at one month after the initial ranibizumab injection. These 8 cases were enrolled in this study.

### Microperimetry

The microperimetry analysis is similar to standard perimetry examination in the macular area within 10 degrees of the fovea and is performed in a dark room. The patient was asked to respond to a light trigger. One of the advantages of this tool is that any refractive error in the patient’s eye can be adjusted for automatically^33^. After obtaining the sensitivity response, this machine acquires a colour fundus photograph then automatically superimposes these 2 sets of data into 1 image (Fig. 1B–D). Given that haemorrhage due to BRVO is more common on the temporal side of the macula^34^, 17 MP3 measurement points on the affected temporal side and the PD on OCTA in a 600-μm square area around that point were measured and the correlation was examined (Fig. 1A–D). The correlation between PD and MP3 at baseline and at 6 and 12 months was examined. The value of MP3 at each time point was recorded for each group of ischemic classification as described above. The transition of the average of all PD and retinal sensitivities was also examined.

### Statistical analysis

The correlation between macular density and retinal sensitivity was analysed using Pearson’s correlation coefficient. The groups were compared using one-way and two-way analysis of variance with the Tukey’s post hoc test. The statistical analysis was performed using GraphPad Prism 8.31 software (GraphPad Software, San Diego, CA, USA). A two-tailed p-value <0.05 was considered statistically significant.

## Supplementary information

Supplementary Figure.
