# Supplementary material for: Prediction of post-treatment retinal sensitivity by baseline retinal perfusion density measurements in eyes with branch retinal vein occlusion
Source: Sci Rep. 2020 Jun 15;10:9614. doi: 10.1038/s41598-020-66708-0 (PMC7295767; doi:10.1038/s41598-020-66708-0)
Supplement: Supplementary file 1 — Supplementary Figure. [file 41598_2020_66708_MOESM1_ESM.docx]

**Prediction of post-treatment retinal sensitivity by baseline retinal perfusion density measurements in eyes with branch retinal vein occlusion**

Soraya Rachima^1^, Kazutaka Hirabayashi^2^*, Akira Imai^2^, Yasuhiro Iesato^2^, Toshinori Murata^2^

^1^Department of Ophthalmology, Diponegoro University, Semarang-Central Java, Indonesia

^2^Department of Ophthalmology, Shinshu University School of Medicine, Nagano, Japan

**Correspondence:** Kazutaka Hirabayashi, MD, PhD

Department of Ophthalmology, Shinshu University School of Medicine, Asahi 3-1-1, Matsumoto, Nagano, 390-8621, Japan

Tel: +81-263-37-2664

Fax: +81-263-32-9448

Email: [khirabay@shinshu-u.ac.jp](mailto:khirabay@shinshu-u.ac.jp)


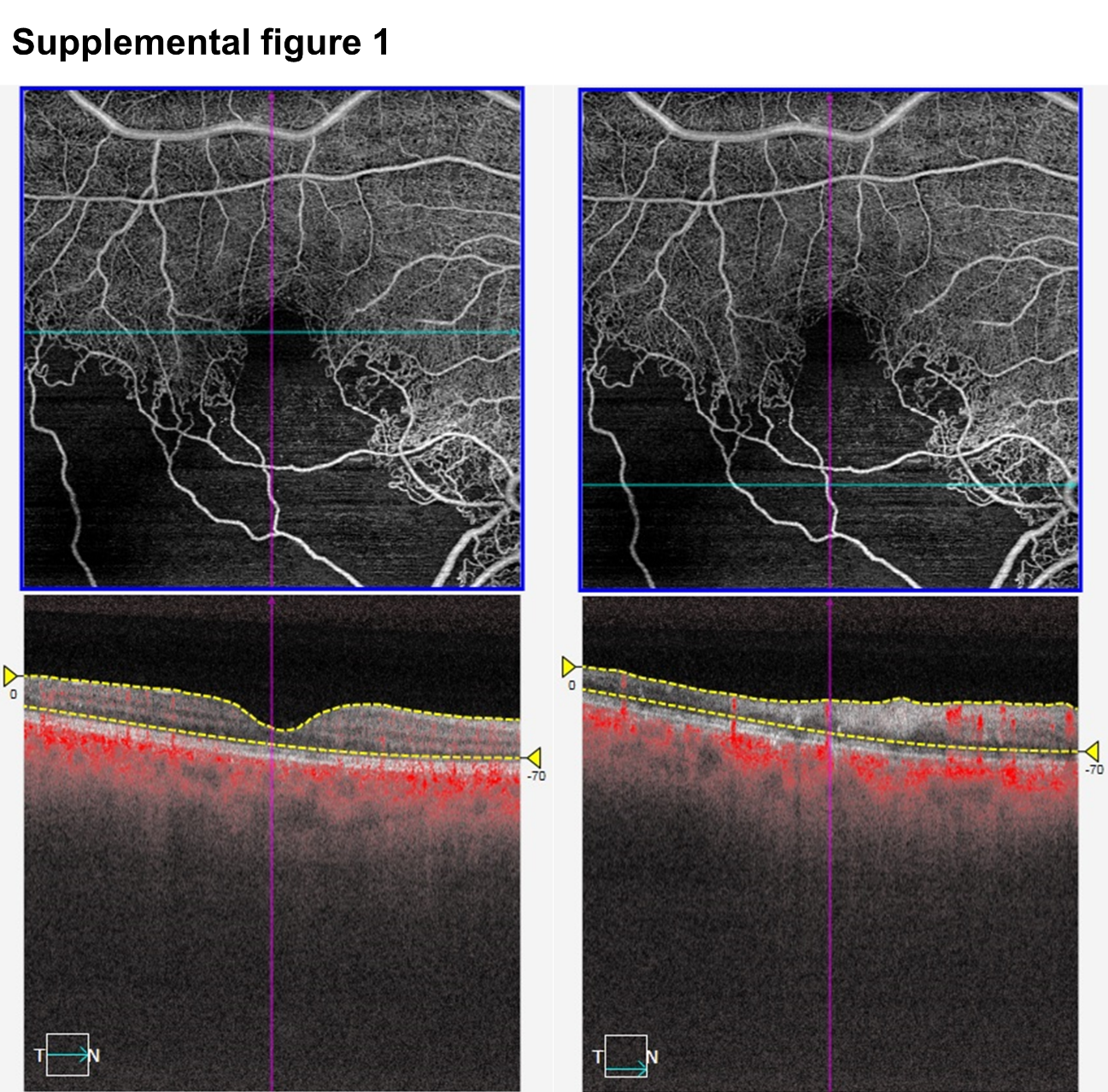


**SUPPLEMENTAL FIGURE LEGEND**

**Figure 1.** En face images of optical coherence tomography angiography (OCTA). Lower: segmentation of OCTA (upper). This study used whole retinal layers (from the internal limiting membrane to above the retinal pigment epithelium surrounded by yellow dot line) (lower). Lower left: B-scan image at the line that crosses the fovea. Many red flow signals are observed in the segmentation. Lower right: B-scan image of the line that crosses non-perfusion area. No red flow signal is observed in the non-prefusion area in the en face image.
